# LIPUS promotes spinal fusion coupling proliferation of type H microvessels in bone

**DOI:** 10.1038/srep20116

**Published:** 2016-02-01

**Authors:** Ximing Xu, Fei Wang, Yahong Yang, Xiaoyi Zhou, Yajun Cheng, Xianzhao Wei, Ming Li

**Affiliations:** 1 Orthopaedic Department of Changhai Hospital, Second Military Medical University, Shanghai, 200433, People’s Republic of China; 2 Department of Physiology, Second Military Medical University, Shanghai, 200433, People’s Republic of China

## Abstract

Low-intensity pulsed ultrasound (LIPUS) has been found to accelerate spinal fusion. Type H microvessels are found in close relation with bone development. We analyzed the role of type H vessels in rat spinal fusion model intervened by LIPUS. It was found LIPUS could significantly accelerate bone fusion rate and enlarge bone callus. Osteoblasts were specifically located on the bone meshwork of the allograft, and were surrounded by type H microvessels. LIPUS could significantly increase the quantity of osteoblasts during spine fusion, which process was coupled with elevated angiogenesis of type H microvessels. Our results suggest that LIPUS may be a noninvasive adjuvant treatment modality in spinal fusion for clinical use. The treatment is recommended for usage for at least one month.

Spinal fusion (arthrodesis) is a commonly accepted procedure for treating patients with spinal disorders such as spinal deformities, tuberculosis, tumor, trauma and other diseases that cause spinal instability^1^,^2^,^3^,^4^,^5^. Arthrodesis is also recommended for degenerative and traumatic diseases of the spine that are associated with severe pain, because mechanical stability is restored by a bony bridge between unstable vertebrae; leading to neurologic recovery and pain relief. Autogenous bone grafts represents the “gold standard” for arthrodesis, since they possess inherent osteoinductive, osteoconductive and osteogenetic properties^6^. However, autogenous bone graft harvesting has often been associated with significant donor-site morbidity, and pseudarthrosis rates may range from 5–40%^7^,^8^. In addition, time to achieve fusion may be prolonged in autogenous bone grafts, or there may be partial fusion or non-union in patients with osteoporosis^9^. Therefore, spine surgeons require an effective method to promote arthrodesis.

Several techniques have been developed to assist spinal fusion. Various grafts are being used such as collagen, calcium phosphate, tricalcium phosphate and demineralized bone^10^,^11^,^12^. In the United States, the FDA has approved the use of human bone morphogenetic protein (hBMP) for posterior spine fusion^13^. Cell therapies include grafts loaded with bone marrow aspirates, mesenchymal stem cells, or platelet-rich plasma^14^,^15^,^16^. Gene therapies that use different vectors to deliver BMP are gaining popularity^17^,^18^. However, the use of these strategies in clinical practice is limited by the lack of efficacy and outcomes data, as well as cost^19^,^20^,^21^.

Recently, clinical trials have found potential therapeutic applications for low-intensity pulsed ultrasound (LIPUS), as a non-invasive adjuvant treatment modality in fracture healing that is associated with no known device-related adverse events^22^,^23^,^24^. Evidence suggests that LIPUS can increase osteoid thickness and bone volume, and is effective for treating delayed unions and non-unions^24^,^25^. Similar to arthrodesis, LIPUS achieves its biological effects by osteogenesis and bone remodeling. Patient compliance with a LIPUS treatment regimen has been reported at 91%^26^. The FDA has approved LIPUS (Exogen;Bioventus Inc., Piscataway, NJ) for treating established non-unions and acute/fresh fractures of the tibia and radius.

Bone formation and bone homeostasis are closely associated with angiogenesis, and studies have shown that endothelial cells (ECs) have an essential role in bone formation^27^,^28^. LIPUS has been found to increase blood flow and angiogenesis during fracture healing^29^,^30^. A novel CD31^hi^Emcn^hi^ endothelial subpopulation (type H ECs) has been identified in bones^27^. Although type H ECs account for <2% of ECs, majority of osteoprogenitors (82% Runx2^+^ cells, 70% Osterix^+^ cells) are selectively positioned around type H endothelium^27^. Furthermore, type H ECs secretes Noggin to regulate osteogenesis^28^.

Currently, the effect of LIPUS on angiogenesis during arthrodesis and the function of type H ECs in arthrodesis remains unknown. This study aims (1) to determine whether LIPUS accelerates spinal arthrodesis by increasing the formation of type H microvessels in bone, and (2) to elucidate the role of type H microvessels in spinal arthrodesis.

## Results

All animals survived until they were sacrificed. One rat in the LIPUS group experienced weight loss after surgery.

### Manual palpation

**Figure 1:**
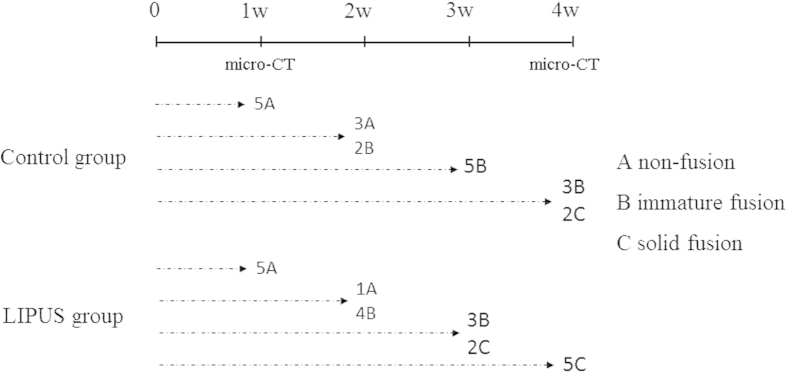
Study design and results of manual palpation.

Manual palpation was performed to assess the fusion status of each specimen. The inter-tester reproducibility was 0.94 (0.89–0.97 for 95% ICC). At one week post-surgery, non-fusion was found in the LIPUS and control groups. At two weeks post-surgery, immature fusion rate in the LIPUS group was two-fold greater than the control group. At three weeks post-surgery, solid fusion rate was 40% in the LIPUS group, while immature fusion rate was 100% in the control group. At four weeks post-surgery, solid fusion rate was 100% in the LIPUS group, while solid fusion rate was 40% in the control group (Fig. 1). These data suggest that LIPUS promoted spinal arthrodesis by accelerating and improving the rate of fusion.

### Radiological assessments

**Figure 2:**
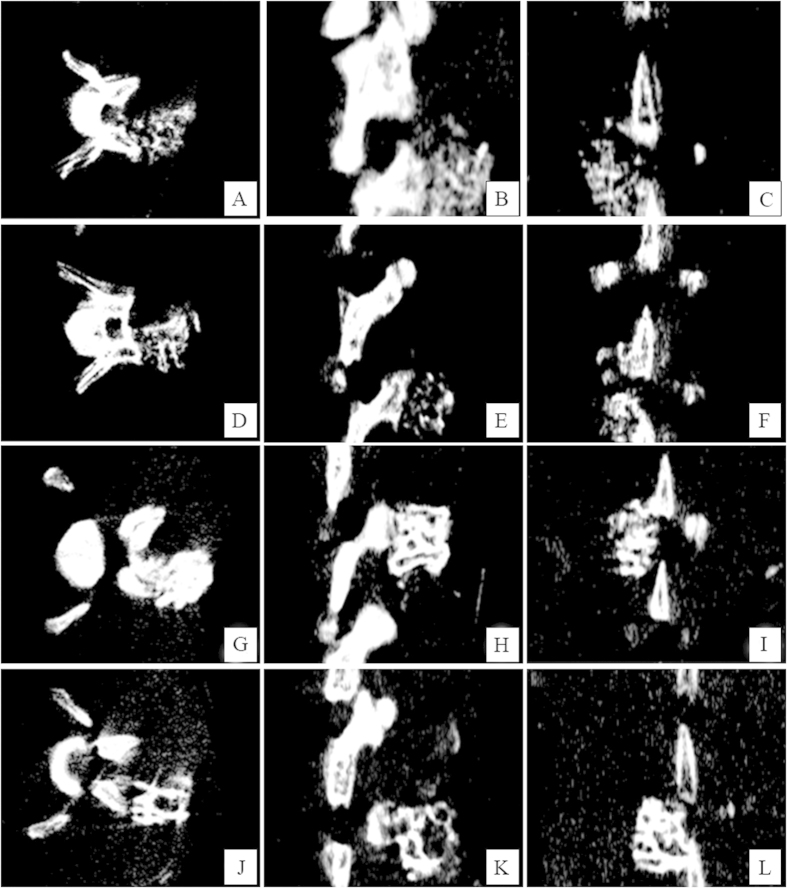
Micro-CT scan. Representative images of fusion masses from coronal (**A**,**D**,**G**,**J**), sagittal (**B**,**E**,**H**,**K**), and axial (**C**,**F**,**I**,**L**) views at one (**A**–**F**) and four (**G**–**L**) weeks post-surgery in the LIPUS group (**A**–**C**,**G**–**I**) and control group (**D**–**F**,**J**–**L**).

Micro-CT analysis was performed on each animal to provide a structural and quantitative evaluation of the spinal fusion (Fig. 2). Complete continuity of the bone graft and transverse process could be seen in the LIPUS group 4 weeks post-surgery. Quantification of total bone volume in fusion masses revealed that mean total bone volume in the LIPUS group was significantly greater compared to the control group 4 weeks after surgery (26.9 ± 0.9 mm^3^ *vs.* 18.0 ± 2.8 mm^3^, *p* < 0.05, respectively).

At one week post-surgery, there were no significant differences between these two groups in any of the parameters (Table 1). At four weeks post-surgery, BV/TV, Tb.N and Tb.Th were significantly greater in the LIPUS group compared to the control group.

### Histological assessments

**Figure 3:**
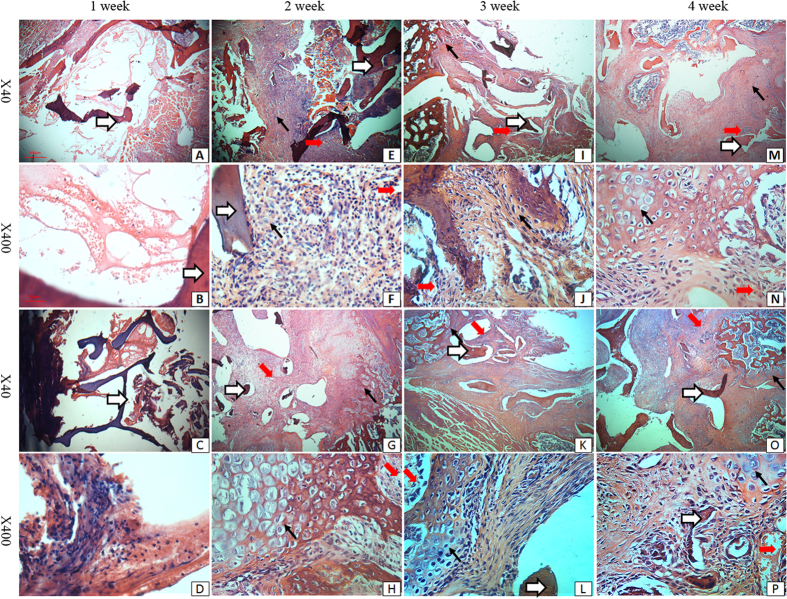
Photomicrographs of the fusion mass in control and LIPUS-treated rats at one week (1^st^ column, A–D), two weeks (2^nd^ column, E–H), three weeks (3^rd^ column, I–L) and four weeks (4^th^ column, M–P) post-surgery. First and third columns, ×40; second and fourth columns, ×400. First and second rows, control group; third and fourth rows, LIPUS group. At four weeks post-surgery, fusion mass in the LIPUS and control groups were composed of a number of chondrocytes, dense fibrous stroma, a large amount of reactive bone, and a number of blood vessels. White arrows allografts; fat red arrows microvessels; thin red arrows chondrocytes. Panel (**A**–**P**) is the same as described in Fig. 3.

At one week post-surgery, no chondrocytes or fibrous tissue could be seen in the fusion bed of the LIPUS or control groups (1^st^ column Fig. 3). At two weeks post-surgery, fibrous tissues could be observed in the fusion site in the control group, while a large number of chondrocytes could be observed in the fusion site in the LIPUS group (2^nd^ column Fig. 3). Many inflammatory cells were scattered in the bone grafts. At three and four weeks after surgery, more chondrocytes and a newly formed bone marrow could be seen in the fusion sites of both groups (3^rd^ and 4^th^ columns Fig. 3). A number of blood vessels were found in the fibrous tissue and bone marrow. Fusion sites were completely invaded by bone tissues intertwined with blood vessels in the LIPUS group (Fig. 3 O/P), while there was less bone tissue in the control group (Fig. 3 M/N). A newly formed bone marrow was observed; and blood vessels were more abundant in grafts in the LIPUS group, compared to grafts in the control group.

### Presence of osteoblasts

**Figure 4:**
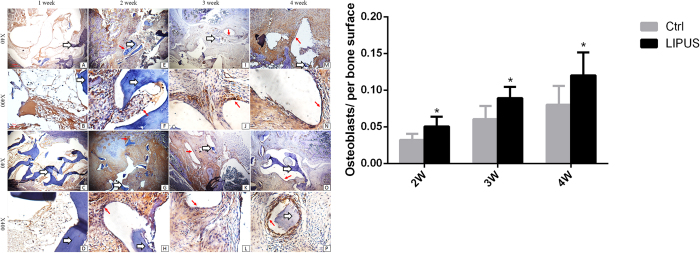
(**A**) Immunohistochemical staining for Osterix and osteoblasts in the fusion mass. The density of Osterix^+^ osteoblasts increased around the bone meshwork of the allograft. Few osteoblasts co-localized with chondrocytes. *White arrows* allografts; *fat red arrows* microvessels; *thin black arrows* osteoblasts. Panel (**A**–**P**) is the same as described in Fig. 3. (**B**) Osterix^+^ osteoblasts were significantly higher in the LIPUS group compared to those in the control group. *P < 0.05.

At one week post-surgery, there were no osteoblasts on grafts in the LIPUS or control groups (1^st^ column Fig. 4A). At two weeks post-surgery, few osteoblasts could be observed in the callus near the transverse process (2^nd^ column Fig. 4A). At three and four weeks post-surgery, a thin layer of osteoblasts could be observed on the allograft in the control group (Fig. 4A K/L, O/P). Conversely, several layers of osteoblasts were observed on the allograft in the LIPUS group (Fig. 4A I/J, M/N). There were more osteoblasts on grafts in the LIPUS group compared to the control group (12.02 ± 3.03 vs. 8.01 ± 2.47, P < 0.01, at four weeks after surgery for LIPUS and control group, respectively; see Fig. 4B and Table 2). Specifically, osteoblasts directly spread and circled the bone meshwork of the allograft, extending as far as the bone meshwork furthest from the interface of the allograft and transverse process where no chondrocytes existed (Fig. 4A JLNP).

### Increased type H blood vessels in the LIPUS group

**Figure 5:**
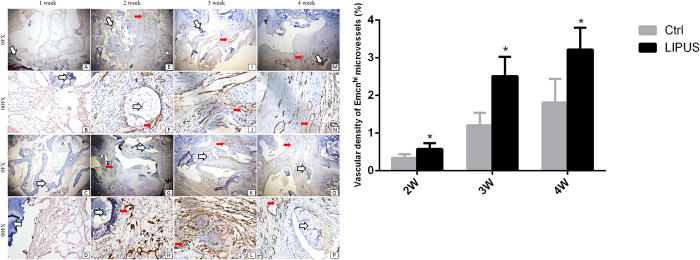
(**A**) Immunohistochemical staining for CD31 and type H microvessels in the fusion mass. The density of CD31^hi^ vessels increased. CD31^hi^ microvessels were spread around the allograft exterior, circling the osteoblasts. Few type H microvessels co-localized with chondrocytes. *White arrows* allografts; *fat red arrows* microvessels. Panel (**A**–**P**) is the same as described in Fig. 3. (**B**) CD31^hi^ microvessels were significantly higher in the LIPUS group compared to those in the control group. *P < 0.05.

Both CD31 and Endomucin were employed to target type H microvessels. At two weeks post-surgery, CD31^hi^ microvessels were present in the LIPUS and control groups (2^nd^ column Fig. 5A). As confirmed by morphometric analysis, the density of CD31^hi^ microvessels was greater in the LIPUS group compared to the control group (Fig. 5A J/L, N/P; Fig. 5B). Mean vascular densities in the LIPUS and control groups were 5.42 ± 0.58% and 3.73 ± 0.87% (P < 0.01) at four weeks after surgery, respectively. In both groups, the majority of CD31^hi^ microvessels spread around the allograft exterior, circling the osteoblasts; and few vessels were observed in the outlying area of chondrocytes. Vessel density of CD31^hi^ microvessels significantly increased from two weeks post-surgery to four weeks post-surgery, and there were significantly more CD31^hi^ microvessels in the LIPUS group compared to the control group (Fig. 5A F/H, J/L, N/P; Fig. 5B; Table 2).

**Figure 6:**
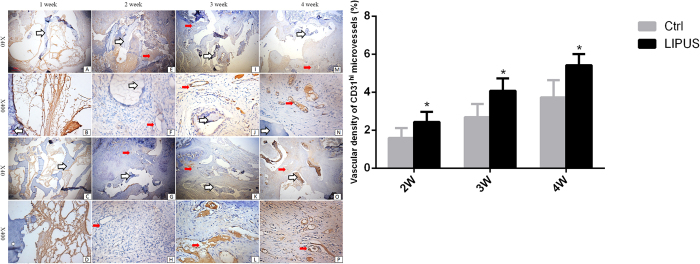
(**A**) Immunohistochemical staining for Endomucin and type H vessels in the fusion mass. The density of Emcn^hi^ microvessels increased. Emcn^hi^ microvessels were spread around the allograft exterior, circling the osteoblasts. Few type H microvessels co-localized with chondrocytes. *White arrows* allografts; *fat red arrows* microvessels. Panel (**A**–**P**) is the same as described in Fig. 3. (**B**) Emcn^hi^ microvessels were significantly higher in the LIPUS group compared to those in the control group. *P < 0.05.

At two weeks post-surgery, Emcn^hi^ microvessels in the callus were sparse in the LIPUS and control groups (2^nd^ column Fig. 6A). At three and four weeks post-surgery, an increased number of Emcn^hi^ microvessels spread around the allograft and in the outlying area of chondrocytes in both groups (Fig. 6A JLNP). Mean vascular densities in the LIPUS and control groups were 3.22 ± 0.56% and 1.81 ± 0.60% at four weeks after surgery (P < 0.01), respectively. There were significantly more Emcn^hi^ microvessels in the LIPUS group compared to the control group (Fig. 6 J/L, N/P; Fig. 6B; Table 2). Emcn^hi^ microvessels were fewer than CD31^hi^ microvessels, but Emcn^hi^ microvessels had the same distribution as CD31^hi^ microvessels.

## Discussion

The results of this study suggest that LIPUS can enhance posterior spinal fusion with allograft in rats. Our histological assessments have shown that osteoblasts were found on the allograft, while chondrocytes were observed near the transverse process. The fusion process was accompanied by the formation of type H microvessels, which were located in the immediate proximity to the osteoblasts.

Ultrasound is a form of mechanical energy that can be transmitted into the body with no thermal effects. LIPUS has been approved by the U.S. FDA for the treatment of fresh fractures and existing non-unions. The study of Zura *et al*. revealed that the healing rate for chronic (1–10 years) non-union fractures in patients (*n* = 767) treated with LIPUS was 86.2%^31^. Another study carried out by Yunkawa *et al*. investigated the effects of LIPUS on spine fusion in patients undergoing single level lumbar interbody fusion^25^. They found that LIPUS could increase fusion rate over a 48-week follow-up period. However, this increase was not statistically significant. In animal studies, LIPUS has significantly increased the fusion rate and fusion mass in spinal fusion models^23^,^24^,^32^. Similarly, the current study revealed that LIPUS could accelerate the fusion process and increase fusion rate in rats, as evidenced by manual palpation and micro-CT, as well as histological analyses. At four weeks post-surgery, fusion rate was 40% in the control group and 100% in LIPUS-treated rats. Bone volume, Tb.N and Tb.Th significantly increased in the LIPUS group, compared with the control group. Furthermore, there was continuity between the allograft and transverse process in the LIPUS group, while a shallow gap existed in the control group. In accordance with our observations, callus in patients treated for fracture healing with LIPUS is larger compared to controls^22^,^23^,^24^,^33^.

In *in vitro* studies, LIPUS has been found to directly affect osteogenic cells and enhance chondrogenesis^34^,^35^. Hui *et al*. and Cook *et al*. observed a mass of chondrocytes at the fusion site in rabbits and dogs, respectively; and suggested that endochondral ossification was the main process involved in spinal fusion^32^,^36^. In the current study, large quantities of chondrocytes were found at the interface of the allograft and transverse process; but were very few in the allograft bone meshwork. In addition, there was significantly more chondrocytes and a newly formed bone marrow in the LIPUS group, compared to the control group. Cook *et al*. found that LIPUS could promote new bone formation and remodeling in a dog spinal fusion model^36^. Hui *et al*. and Wang *et al*. reported similar LIPUS promoting effects during spinal fusion in other bone grafts^32^,^37^.

Notably, we found that the number of osteoblasts surrounding the bone meshwork throughout the allograft increased with time after surgery in both the control and LIPUS groups. These observations were in accordance with our radiological findings. Interestingly, osteoblasts were not localized around chondrocytes, and were positioned on the allograft meshwork instead. The density of osteoblasts on the bone meshwork significantly increased in the LIPUS group, compared to the control group. Specifically, osteoblast did not appear at one week after surgery in both control group and LIPUS group. More osteoblasts were observed at two weeks post-surgery in the LIPUS group. And there was much more osteoblasts at four weeks post-surgery compared with at 2 or 3 weeks post-surgery. Based on these findings, we speculate that osteoblast-induced bone formations play a central role in spinal fusion, rather than endochondral ossification. Zhuo *et al*. used porous hydroxyapatite blocks as a graft for spinal fusion, and found that LIPUS increased the number of osteoblast-like cells in the porous blocks, as evidenced by a scanning electron microscope^38^. However, they did not describe the distribution of osteoblasts. There have been few reports of clinically relevant data describing the effects of LIPUS on temporal changes in osteoblast frequency, and spatial distribution with time during spinal fusion. This is because most previous studies used a collagen sponge for the graft, which might induce a different pathological fusion process, compared to that associated with a freeze-dried allograft^32^,^36^,^37^. In *in vitro* studies, LIPUS increased the proliferation and maturation of osteoblasts, as well as the formation of the extracellular matrix^34^,^35^.

Evidence has suggested that angiogenesis is associated with osteogenesis, providing oxygen, stem cells, and growth factors that promote the osteogenic process. In this study, we aimed to investigate the role of type H microvessels in an osteogenic microenvironment^27^,^28^. Previous reports have shown that decreased numbers of CD31^hi^Emcn^hi^ ECs are associated with decreased osteogenic activity and osteoporotic changes^27^, and that pre-osteoblast cells are selectively positioned around type H microvessels. The study of Ralf *et al*. demonstrated a critical role for the Notch signaling pathway in angiogenesis. They revealed that Noggin, which is regulated by Notch, is secreted by type H microvessels to promote the proliferation of osteoprogenitor cells^28^. Therefore, we investigated whether type H microvessels are involved in bone formation and remodeling during spinal fusion. Immunostaining has shown that the density of CD31^hi^Emcn^hi^ microvessels increased during fusion. Significantly, more type H microvessels were found in the LIPUS group compared to the control group.

Solid fusion rate (determined by manual palpation), mass of the bone callus (determined by radiological assessment), the amount of bone tissues and number of osteoblasts in the fusion mass (determined by histological assessments), and the density of type H microvessels observed during the fusion process were greater in the LIPUS group compared to the control group. It was not until at two weeks post-surgery that type H microvessels were observed in both control and LIPUS groups. The quantity of type H microvessles increased as time going. At 4 weeks post-surgery, more type H microvessles were found in the fusion area compared with at 2 or 3 weeks post-surgery. Meanwhile, larger quantities of type H microvessels were in the LIPUS group compared with the control group. Importantly, the density of type H microvessels located in immediate proximity to the osteoblasts increased in parallel with the number of osteoblasts as fusion progressed. Taken together, these data suggest that type H microvessels play an essential role in the promotion of bone remodeling by LIPUS during spinal fusion. And it was not until two weeks treatment that LIPUS had effect in increasing quantity of osteoblasts as well as angiogenesis of type H microvessels.

Park *et al*. found that the increased rate of spinal fusion and greater bone volume was associated with denser vascularity in COMP-angiopoietin 1-treated rats that underwent bilateral posterior and posterolateral arthrodesis with allograft, compared to rats treated with bovine serum albumin^39^. These newly formed vessels were positioned in the primary mineralizing area where osteogenesis was actively occurring. Liu *et al*. studied the effect of calcitonin on lumbar spinal fusion in a rabbit model, and found that calcitonin could enhance lumbar spinal fusion by increasing the expression of genes involved in osteogenesis and angiogenesis including collagen I, BMP-2, insulin-like growth factor-1, and vascular endothelial growth factor (VEGF)^40^. The study of Street *et al*. revealed that exogenous VEGF enhances angiogenesis, ossification, and new bone formation in a mouse model of fracture repair and a rabbit critical size defect model^41^. Treatment of mice with a VEGF receptor antagonist inhibited osteogenesis and fracture healing, while preventing angiogenesis. These data suggest that angiogenesis is indispensable for osteogenesis during spinal fusion. In *in vitro* studies, LIPUS causes osteoblasts to migrate and secrete VEGF and other growth factors^42^,^43^, and promotes the migration of ECs^44^. An *in vivo* study carried out by Cheung *et al*. revealed that LIPUS could enhance callus formation and angiogenesis during fracture healing in a rat model, and VEGF expression was significantly increased four weeks after surgery^45^. In accordance with these data, Azuma *et al*. found that LIPUS could accelerate fracture healing via angiogenesis in rats^46^. Lu found that LIPUS could improve bone-joint healing through the regulation VEGF expressions in the early phase, and subsequent chondrogenesis in rabbits^47^. Changes in VEGF expressions preceded endochondral ossification and bone remodeling. These studies have shown that LIPUS may affect the function of ECs, indicating that LIPUS may induce an increase in type H microvessels and promote spinal fusion.

This study is associated with several limitations. First, the mechanical strength of the fusion mass was not evaluated, because a single-level fusion procedure was employed in this study; which did not allow the measurement of the joint flexion. Second, the rat spine is small, which makes it difficult to accurately measure the fusion mass. However, manual palpation was used, which has been proven to be effective in fusion rate evaluation^39^. In the current study, manual palpation results were in accordance with micro-CT findings and histological analyses. Third, the mechanism by which type H microvessels regulate bone remodeling during spinal fusion was not investigated, because the microenvironment of allograft fusion differs from normal osteogenesis.

## Conclusions

In conclusion, LIPUS can accelerate spinal fusion and increase bony mass in a rat spinal fusion model. This process is coupled with chondrogenesis, increased osteoblasts, and angiogenesis. Specifically, LIPUS significantly increased the proliferation of osteoblasts and type H microvessels. The main process of spinal fusion is osteoblast-induced bone formation, which process is coupled with angiogenesis of type H microvessels. LIPUS is recommended for enhancing spinal fusion for treatment of at least four weeks post-surgery. Further research is required to elucidate the origin of the osteoblasts that are formed during spinal fusion, and the factors that determine the spatial distribution of type H microvessels relative to osteoblasts.

## Methods

### Experimental design

Single level unilateral posterior spinal arthrodesis was evaluated in 40 male Sprague–Dawley rats. LIPUS was administered to 20 rats on the surgical area for 20 minutes per day post-surgery (LIPUS group), while the remaining animals received sham treatment (control group). Five animals were sacrificed at each of the following time points: one, two, three and four weeks post-surgery. Manual palpation and micro-computed tomography (micro-CT) scanning was performed on each animal after they were sacrificed at one and four weeks post-surgery to evaluate spinal fusion. The fusion mass was analyzed histologically and immunohistochemically to investigate the pattern of the fusion. This study was approved by the Ethical Committee of Changhai Hospital. All animals were handled strictly according to the Good Animal Practice requirements of the Animal Ethics Procedures and Guidelines of the People’s Republic of China.

### Animal model and surgical procedure

Forty male Sprague–Dawley rats (age, 12 weeks; weight, 300–350 g) were kept at 22 °C with free access to water and food. Anesthesia was induced and maintained with isoflurane (0.5% to 2%) and oxygen using a coaxial nose cone. After shaving the surgical site, a posterior lateral incision was made over the lumbar spine. The transverse process of the L4 vertebra was exposed by bluntly splitting the back muscles referencing the iliac crest. Once exposed, the transverse process was decorticated with an electric bur until shallow bleeding was observed. Then, a demineralized freeze-dried bone allograft (Aorui Biological Material Co., Ltd., Shanxi, China) was implanted on the decorticated fusion beds of the L4 transverse process. Finally, fascia and skin were closed with interrupted sutures. Postoperative antibiotics were administered intramuscularly for two days (cefuroxime, 0.5 mg/kg). None of the animals died before euthanasia. Twenty-five preliminary trial operations were performed to confirm the reliability of this model. The surgery was all performed by XMX. Animals were randomly allocated to LIPUS group or Control group by XZW.

### LIPUS device and treatment

Rats were randomly divided into two groups (*n* = 20 each), and all animals were anesthetized. LIPUS was initiated in use from 3rd day post-operatively. Rats in the LIPUS group were treated for 20 minutes per day (five days a week) with an Exogen 2000^+^ device (Bioventus Inc., Piscataway, NJ) with the following specifications: 30 ± 5.0 mW/cm^2^ spatial average and temporal average incident intensity, 1.5-MHz pulsed frequency, 200-μs pulse duration, 1.0-kHz repetition rate. Rats in the control group were treated with the same device for the same duration and number of days, but with no power. The transducer head was centered over the fusion site. Coupling gel was carefully smeared to ensure contact between the transducer head and skin. LIPUS treatment was conducted by XMX and XYZ.

### Manual palpation assessment

Fusion mass at the bone-grafted segment was assessed by manual palpation *ex vivo*^48^ The lumbar spine was gently palpated immediately after death by two assessors (FW and YHY), who were blind to group allocation. Disagreement was resolved by a third rater ML. Bridging bone formation between the graft and the L4 transverse process was evaluated. Fusion status of each specimen was graded based on the following categories: solid union, defined as no motion detected; immature union, defined as bony continuity with slight motion; and non-union, defined as wide motion detected. Fusion rate was defined as the percentage of solid or immature unions. Manual palpation is an established method for assessing the stiffness of the fusion mass in rats^49^,^50^, since mechanical stiffness of the spine segment is difficult to measure due to the small size of the grafted segment and complicated shape of the rat spine. Inter-tester reproducibility was tested via calculating intra-class coefficient (ICC).

### Micro-computed tomography scanning

Using a CT scanner (GE Healthcare Bio-Sciences Corp., Piscataway, NJ, USA), micro-CT was performed in the axial plane to evaluate the calcified bone graft. Scans were initiated from the pelvis to the L1 vertebral body cranially in 13-μm sections. Microstructural indices were measured using MicroView (GE Healthcare Bio-Sciences Corp., Piscataway, NJ, USA). Bone volume/tissue volume (BV/TV), trabecular thickness (Tb.Th), trabecular number (Tb.N), and trabecular separation (Tb.Sp) were calculated.

### Histologic evaluation

After removal, L4-L5 transverse process fusion area were fixed with 4% paraformaldehyde for 24 hours. Then the specimens were decalcified in 5% nitric acid for 3 days, washed in distilled water, and embedded in paraffin^51^. Transversal sections (5 μm) were obtained from the midline of the transverse process area. Sections were stained with hematoxylin and eosin (H&E), and observed under light microscopy.

### Immunohistochemical staining for osteoblasts and blood vessels

Osteoblasts and type H microvessels were immunostained. Briefly, sections were rinsed in phosphate buffered saline to remove traces of the fixative, and were incubated in normal bovine serum for two hours at room temperature to block nonspecific binding of antibodies. Then, sections were incubated with goat polyclonal Osterix, CD31, and Emcn primary antibodies (diluted at 1:50; Santa Cruz, CA, USA) for 24 hours at 4 °C. After rinsing in phosphate buffered saline, fluorescein isothiocyanate conjugated bovine anti-goat secondary antibody (diluted 1:100; Santa Cruz, CA, USA) was applied for one hour at 37 °C. The sections were mounted after a final rinse, and examined under an Olympus eclipse 80i (Olympus, Japan) fluorescence microscope equipped with a mercury lamp and filter system. In histological analysis, the transverse process was first to be identified. Areas between transverse process and superior allograft were the interested fusion areas. Three sections were obtained in each specimen for statistical analysis. Osteoblast numbers or vascular densities in each section were acquired by calculating the means of five randomly captured views (×400 magnification). Osteoblast number per bone surface (Ob/BS) was calculated^52^. Mean vascular densities was calculated as (CD31^hi^ or Emcn^hi^ vascular area/total image area in ×400 magnification) ×100%. Analysis of images involved ImagePro Plus 5.02 (Media Cybernetics).

### Statistical analysis

SPSS 16.0 was used for statistical analyses. Differences in fusion status between groups were analyzed by Chi-squared test. Micro-CT and histomorphometric data are expressed as mean ± standard deviation (SD). The density of type H microvessels across various healing stages in the fusion mass was compared by one-way analysis of variance (ANOVA) for repeated measurements. Differences of osteoblast numbers and MVD between the control group and LIPUS group were analyzed by t test. *P* < 0.05 was considered statistically significant.

## Additional Information

**How to cite this article**: Xu, X. *et al*. LIPUS promotes spinal fusion coupling proliferation of type H microvessels in bone. *Sci. Rep.* **6**, 20116; doi: 10.1038/srep20116 (2016).

## Figures and Tables

**Table 1:** Fusion mass parameters evaluated by micro-CT scanning.

**Group**	**Control**	**LIPUS**	**Control**	**LIPUS**
	**One week post-surgery**		**Four weeks post-surgery**	
BV/TV (%)	27.2 ± 2.3	27.3 ± 2.3	33.4 ± 1.5	42.6 ± 2.9^*^
Tb.Th (um)	30.9 ± 1.1	31.6 ± 2.0	32.1 ± 1.5	38.7 ± 1.8^*^
Tb.N (1/um)	6.7 ± 0.4	7.6 ± 0.7	8.6 ± 0.4	12.3 ± 1.2^*^
Tb.Sp (um)	111.4 ± 17.0	101.1 ± 14.3	68.5 ± 4.5	45.5 ± 2.3^*^

*P < 0.01

CT, computed tomography; LIPUS, low-intensity pulsed ultrasound; BV/TV, bone volume/tissue volume; TB.Th, trabecular thickness; Tb.N, trabecular number; Tb.Sp, trabecular separation.

**Table 2:** Quantity analysis of osteoblasts and type H microvessels.

	**2 Week (N = 15)**		**3 Week (N = 15)**		**4 Week (N = 15)**	
	**Control**	**LIPUS**	**Control**	**LIPUS**	**Control**	**LIPUS**
Osteoblasts (Ob/ per bone surface)	3.22 ± 0.80	5.04 ± 1.31^*^	6.06 ± 1.73	8.92 ± 1.49^*^	8.01 ± 2.47	12.02 ± 3.03^*^
CD31^hi^ microvessels MVD(%)	1.6 ± 0.51	2.43 ± 0.52^*^	2.69 ± 0.66	4.07 ± 0.63^*^	3.73 ± 0.87	5.42 ± 0.58^*^
Emcn^hi^ microvessels MVD(%)	0.34 ± 0.10	0.58 ± 0.15^*^	1.2 ± 0.32	2.51 ± 0.5^*^	1.81 ± 0.60	3.22 ± 0.56^*^

*P < 0.01. Ob, osteoblast. MVD, mean vascularity density.
